# Congenital Retinal Macrovessel; Optical Coherence Tomography Angiography Features

**DOI:** 10.18502/jovr.v18i2.13191

**Published:** 2023-04-19

**Authors:** Ramin Nourinia, Kiana Hassanpour

**Affiliations:** ^1^Ophthalmic Research Center, Research Institute for Ophthalmology and Vision Science, Shahid Beheshti University of Medical Sciences, Tehran, Iran; ^2^Department of Ophthalmology, Labbafinejad Medical Center, Shahid Beheshti University of Medical Sciences, Tehran, Iran

##  PRESENTATION

Congenital retinal macrovessel (CRM) was first described by Mauthner in 1869 and was further classified by Brown in 1982 as an aberrant retinal vessel that crosses the horizontal raphe in the macular region. Abnormal fetal development between the 15 ${}^{th}$ and 16 ${}^{th}$ weeks of embryogenesis is thought to cause CRM.^[1]^

A 60-year-old otherwise healthy woman presented to our clinic complaining of gradual decreased vision in the right eye. The best-corrected visual acuity was 20/40 refracting with +1.00 –1.00 × 180. Complete ocular examination revealed no pathologic findings except mild nuclear sclerosis cataract in both eyes. Fundus examination revealed an aberrant vessel crossing the horizontal raphe in the macular region in the right eye.

**Figure 1:**
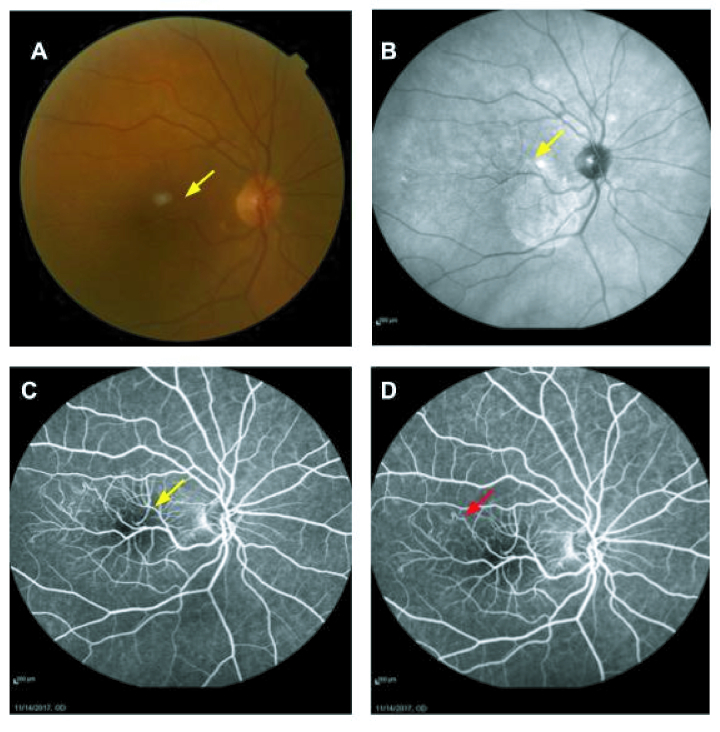
(A) Fundus photograph of the right eye showing a dilated, retinal vein moving toward the macula and crossing the horizontal raphe, suggestive of a venous congenital retinal macrovessel (CRM). (B) Corresponding infrared image shows the aberrant vessel where its first and second branching cross the horizontal raphe in the macular area. Abnormal telangiectatic vessels are seen at the end of the branches. (C & D) Fundus fluorescein angiography confirmed a venous CRM with draining venules crossing the horizontal raphe (yellow arrow), and telangiectatic capillaries are seen at the distal part of the branches (red arrow).

Figure 1 shows the fundus photograph, infrared image, and fluorescein angiography of the right eye. An aberrant retinal vessel that originated from the inferotemporal part and moved toward the superior part of the macula is evident in all images. Telangiectatic capillaries are also seen at the distal part of the branches.

**Figure 2:**
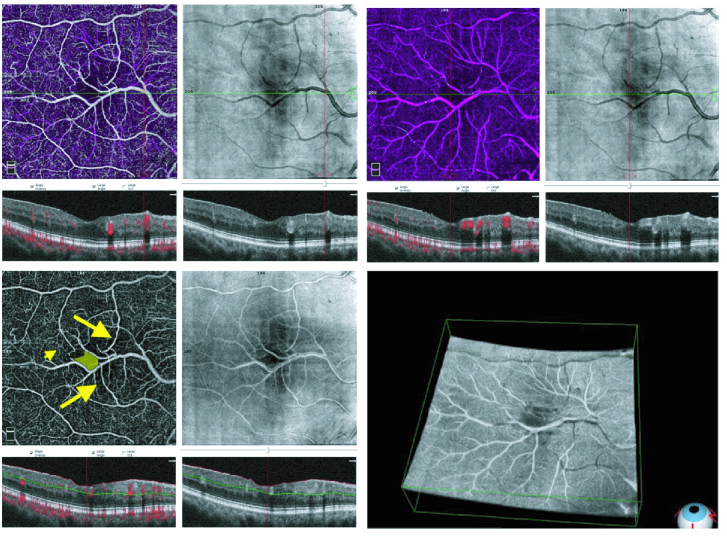
C-scan image of optical coherence tomography angiography shows a large aberrant vessel crossing the center of the macula, and an unusual shape of the foveal avascular zone. Large vessels that connect the superior and inferior main vessels to the aberrant vessel are also evident (large arrows). Similarly, small vessels connect the superior and inferior vessels to the aberrant vessels (in both deep and superficial layers; small arrowhead). The B-scan image of the optical coherence tomography angiography shows the sections of the aberrant vessel adjacent to the foveal center.

The optical coherence tomography angiography (OCTA) illustrates an abnormal aberrant vessel in the inferior part of the macula crossing the center. There are also large and small vessels making connection between the aberrant vessel and the superior and inferior macular main vessels in both superficial and deep layers [Figure 2]. The images demonstrating outer segments shows that the aberrant vessel did not penetrate deeper than the outer nuclear layer (ONL). Flow-overlay images show the presence of blood flow in these vessels. Besides, the unusual and distorted shape of the foveal avascular zone (FAZ) is also evident [Figure 2].

This is an open access article distributed under the Creative Commons Attribution License, which permits unrestricted use, distribution, and reproduction in any medium, provided the original work is properly cited.

##  DISCUSSION

CRMs are usually unilateral incidental findings without any visual symptoms which are most possibly of venous origin. However, the association between CRMs and many other retinal pathologies including central serous chorioretinopathy (CSCR), retinal hemangioma, microaneurysm, and rhegmatogenous retinal detachment have been reported.^[2]^ Moreover, other vascular abnormalities including brain venous malformations are also reported in association with CRMs. Therefore, neuroimaging is recommended to find any malformation in brain vessels.^[3]^

Multimodal imaging of a CRM provides a better understanding of the nature of aberrant vessels. OCTA allows for the separation of deep and superficial capillary plexus which results in a better demonstration of the levels and paths of aberrant vessels.^[2,4]^ In our case, we manually adjusted the segmentation of the OCTA. The dominance of these aberrant vessels in the deep capillary plexus (DCP) might confirm its nature to be venous in origin as DCP is considered the layer more responsible for venous drainage.^[5]^ Another advantage of OCTA is that concurrent vascular pathologies like retinal hemangioma or choroidal neovascularization can be simultaneously ruled out.

##  Financial Support and Sponsorship

None.

##  Declaration of Patient Consent

The authors certify that they have obtained all appropriate patient consent forms. The patient has given her consent for the use of images and other clinical information to be reported in the journal. The patient understands that her name and initial will not be published and due efforts will be made to conceal her identity, but anonymity cannot be guaranteed.

##  Conflicts of Interest

The authors declare no conflict of interest.
